# Experiences, Perceptions and Attitudes Toward Bullying Among School-Going Adolescents: A Cross-Sectional Study from South India

**DOI:** 10.1007/s40653-024-00631-8

**Published:** 2024-03-26

**Authors:** Nayana Narayanan Nedumpully, Samir Kumar Praharaj, Shweta Rai

**Affiliations:** 1 Present Address: Department of Psychology, St Aloysius College, Elthururth, Thrissur, Kerala India; 2 Department of Psychiatry, Kasturba Medical College, Manipal, Manipal Academy of Higher Education, Manipal, Karnataka 576104 India; 3 Department of Clinical Psychology, Manipal College of Health Professions, Manipal Academy of Higher Education, Manipal, Karnataka 576104 India

**Keywords:** Bullying, Adolescents, School, Experiences

## Abstract

Bullying victimisation affects an estimated 30% of individuals worldwide. While the prevalence and risk factors of bullying have been studied in India, comprehensive research on the phenomenon of bullying itself remains scarce. Our objective was to study the experiences, perceptions, and attitudes towards bullying among seventh to ninth-grade students. The study included all seventh to ninth graders (*N* = 205) from two schools in the Udupi district of South India. To collect information on bullying, we used the *Bully Survey - Student Version* with appropriate modifications for our context. The mean age of the participants was 13 (1.05) years, with 58% being females. Our findings showed that almost half of the students had bullying roles. Students attending private schools and residing in urban areas were more likely to be victims, bullies, and bully-victims. Seventh and eighth graders experienced higher rates of bullying, whereas ninth graders were more likely to engage in bully behaviours or be bully-victims. Verbal bullying, including name calling, playing jokes, and making fun of others, was more prevalent. No gender differences were observed in verbal or physical bullying. School teachers, staff, and parents were unaware of bullying incidents almost half of the time. Anti-bullying programs should consider these aspects of bullying to be effective.

## Introduction

Bullying represents a form of interpersonal violence that occurs during various stages of a child’s development, especially in adolescence – a period marked by significant transitions in the physical, psychological, and social realm, making them vulnerable in certain aspects of relationships (Ferrara et al., 2019). Understanding the complex relationship dynamics in this age group is difficult but a prerequisite for their wellbeing (Gómez-López et al., 2019). Bullying is a social issue that needs to be carefully addressed due to its sensitive and chronic nature. It is an age-old phenomenon worldwide, but relatively less studied in the East. A recent global estimate on bullying prevalence conducted in 83 countries provided a 30% pooled prevalence of bullying victimization, irrespective of income status (Biswas et al., 2020).

Daniel Olweus, pioneer of bullying research has systematically studied the nature and prevalence of school bullying in the 1970s and has identified the critical elements of bullying: an imbalance of power, the intent to harm, and repetition over time, which clearly distinguishes it from other acts of aggression or violence. Bullying may take many forms, ranging from seemingly minor acts such as name-calling and teasing to more severe forms such as physical, verbal or even sexual attacks, or cyberbullying (Radliff et al., 2018). Such experiences make the individual susceptible to various negative emotional, behavioural, and mental health outcomes (Takizawa et al., 2014; Wolke & Lereya, 2015; Evans-Lacko et al., 2017). These outcomes may include internalizing/externalizing problems, bodily disturbances, and even suicide attempts when compared to those who have not experienced bullying (Swearer, 2001; Hinduja & Patchin, 2010; Kelly et al., 2015; Eastman et al., 2018; Menken et al., 2022).

Most studies conducted in India (Thakkar et al., 2021) have focused on examining the prevalence, forms, and risk factors of bullying, rather than the phenomenon itself, i.e. the bullying profile, experiences, and attitudes towards bullying in those in various bullying roles. This appreciates why bullying research is important, however, bullying cannot be prevented unless we learn about the experiences of those who are involved. Many studies have examined the proportion of students in different bullying roles (Kshirsagar et al., 2007; Malhi et al., 2014; Malik & Mehta, 2016; Patel et al., 2017; Ramya & Kulkarni, 2011), but did not provide a detail of their profiles. An individual may assume different roles in a bullying scenario and there are chances that it may overlap i.e., those who are bullied in a situation may act as a perpetrator in other situations, and vice versa, thus, acknowledging multiple roles of the individual. Only one study (Ramya & Kulkarni, 2011) has explored the setting and participants involved in bullying incidents. Understanding the prevalent forms of bullying, locations where it is happening, persons involved, and perceived reasons for bullying can assist educators, researchers, and policy makers in designing effective interventions for preventing bullying. Specifically, cyberbullying, occurring in online spaces, may require different monitoring and prevention approaches (Gabrielli et al., 2021; Touloupis & Athanasiades, 2022; Santre, 2022).

Evaluating the responses of teachers and other staff members to bullying incidents from the students’ perspective is important in understanding the role of school policies aimed at tackling bullying (Salmivalli & Voeten, 2004; Denny et al., 2015). Collaborating with parents is a crucial step in anti-bullying efforts, however, the extent of communication between students and parents regarding bullying incidents happening to them is least explored in previous studies. Furthermore, determining adolescents’ pro-bullying attitudes, which support bullying behaviour, is vital in understanding their potential inclination towards such behavior, as these attitudes can have powerful influence the individuals’ thoughts and actions in situations (Pickens, 2005). Adolescents exhibiting positive attitudes towards bullying tend to engage in increased bullying behavior (Salmivalli & Voeten, 2004; van Goethem et al., 2010). All these components serve to understand the phenomenon better and to design individualised bullying prevention programs for schools. Thus, our study aimed to explore the experiences, perceptions, and attitudes towards bullying among seventh to ninth graders. The specific objectives of our study were: (1) characterizing the bullying roles among adolescents; (2) delineating the various types of bullying; (3) examining gender differences in bullying; (4) profiling adolescent bullying, including locations, participants, reasons for bullying, school awareness and response to bullying; and (5) assessing attitudes towards bullying.

## Method

### Participants

This cross-sectional study was conducted at the Department of Psychiatry, *(anonymized)*, a coastal city in South India, from July to November 2019. The study was approved by the Institutional ethics committee *(anonymized)* and the protocol was registered under CTRI *(anonymized)*. Permissions were sought from the school principals and the Deputy Director of Public Instruction, Udupi, Karnataka, to approach the class. Sample size estimate for proportions with 30% prevalence of bullying victimisation, with a 5% precision and a 95% confidence interval for a large population is 322. However, the study included all seventh to ninth graders (*N* = 205) from two schools, one English medium private school and one Kannada medium government state-run school. The participants were required to have the ability to read and write English/Kannada. Their mean age was 13 (*SD* 1.05) years, and 58% were females. Socio-demographic profile of the participants is presented in Table 1. Informed consent from the parents and assent from the students were obtained prior to the study.

**Table 1:** Demographic profile of the participants (*N* = 205)

Variable		M/n	SD/%
Age		13.0	1.1
Gender	Male	86	42.0
	Female	119	58.0
School	Private	116	56.6
	Government	89	43.4
Grade	VII	87	42.4
	VII	63	30.7
	IX	55	26.8
Residence^a^	Rural	88	42.9
	Urban	117	57.0
Kannada (native)	Yes	116	56.6
	No	89	42.9
Bullying roles^b^	Victim	59	28.8
	Bully	13	6.3
	Bully-Victim	24	11.7
	No role (does not experience victimization/bullying)	109	53.2
Total BAS^c^		26.7	8.0

Note. ^a^The residence category used as per the Census India, 2011; Rural unit included villages and areas which are not categorized as urban units, and Urban units included towns, municipalities, cooperation, cantonment board, statutory town etc

^b^Roles are classified based on self-report, and no role includes bystanders

^c^Total of Bullying Attitude Scale

### Measures

Socio-demographic information and clinical details were collected using a proforma designed for this study. The *Bully Survey - Student Version* (BYS-S, version 08/2016) developed by Susan Swearer was used to collect details of bullying. This survey consists of four-parts and 46-questions that ask students about their experiences, perceptions, and attitudes toward bullying. Each part of the survey begins with a definition of bullying: “Bullying happens when someone hurts or scares another person on purpose and the person being bullied has a hard time defending himself or herself. Usually, bullying happens over and over” (Swearer, 2001). The survey is divided into 4 parts – A, B, C, D – each part probes into different aspects, when they were bullied, observed bullying, bullied other students during the previous year, and attitude towards bullying, respectively. Participants were instructed to skip sections if they have not been bullied, witnessed bullying, or bullied others. The final section of the survey measures attitudes toward bullying through *Bully Attitudinal Scale* (BAS) that contains 15-items. Each item of the scale describes attitudes towards bullying and prosocial attitudes. Participants rated the extent of their agreement with each item on a five-point scale: 1 = “Totally False” to 5 = “Totally True.” Higher total scores on BAS indicate stronger pro-bullying attitudes. Based on the screening questions provided in the survey, participants self-identified their bullying roles as: (a) victim, (b) bully, (c) bully-victim, or (d) no role. In our sample, the internal consistency of the 15 items of BAS as measured by Cronbach’s α was 0.705.

The Survey was adapted with specific changes in wording. This involved removal of unfamiliar terms such as ‘homeroom,’ ‘locker room,’ ‘gym’ (questions q2a, 16a, 25a), replacing them with ‘assembly hall,’ which denotes a large space in school for regular meetings. Additionally, terminologies like ‘IMing’ (questions q2b, 16b, 25b) was reworded to ‘instant messaging’ for easy understanding. The survey underwent a translation following the WHO protocol. Initially, the questionnaire was translated into Kannada by a bilingual mental health professional proficient in both English and Kannada. Subsequently, another bilingual individual, unaware of the original English version, performed a back translation of the Kannada questionnaire. Any discrepancies or ambiguities in meaning were resolved through discussions and consensus among the translators to produce a final translated version. The revised version underwent evaluation by subject experts to establish face validity. The final version, agreed upon after this rigorous process, was included in the study.

### Procedure

Two schools, one with Kannada medium and one with English medium instruction, were selected through convenience sampling with the permission from the respective school principals. Participant information sheets and informed consent forms, available in both Kannada and English, were distributed to parents through the students. These forms were collected back with the help of class teachers. Parents were given the opportunity to contact the researcher for any clarification or queries about the study, before consenting. The study objectives were explained to the participants and were given an opportunity to discuss their concerns and queries. The survey was administered during a free hour on a regular school day. The data collection was overseen by the first author, a PhD scholar. While most students completed the survey independently, some required additional help on some items, which the researcher clarified as needed. Throughout the study, anonymity of the participants was strictly maintained. Most students completed the survey within the given time. The teacher-in charge of each class helped in maintaining discipline and silence throughout the session.

### Statistical Analysis

The data analysis was carried out using IBM SPSS version 27. Descriptive statistics, using means, frequency, and percentages were used to summarize the data. To explore the association between the four bullying roles (victim, bully, bully-victim, and no role) and various demographic variables such as gender, school type, grade, residence, and native language, Chi-square tests of independence were performed. Normality of data was examined through the Shapiro-Wilk test and plotting histograms. Due to non-normal distribution across bullying roles and a relatively small sample size, the Mann-Whitney U test was utilized to examine differences in physical and verbal bullying between boys and girls. To evaluate variations in pro-bullying attitudes across bullying roles among different grades, a one-way ANOVA was conducted. Independent sample t-tests were used to compare pro-bullying attitude scores across gender, school type, residence, and native language. Post hoc Tukey tests were used to compare the mean difference between groups. All *p* values < 0.05 (2-tailed) were considered statistically significant.

## Results

### Bullying Roles

In our study, 47% of the students had bullying roles, while 53% reported no experience of bullying or victimisation. The relationship between bullying roles and various demographic variables is summarized in Table 2. The analysis showed significant association between bullying roles and school type (χ^2^ (3) = 25.9, *p* < .001). Students from private school were more likely to be victims, bullies, and bully-victims, whereas those in government schools were more likely to be in ‘no role’ group. There was a significant relationship between bullying roles and grade (χ^2^ (6) = 27.8, *p* < .001). Most of the students in ‘no role’ group were in grade VIII, followed by grades IX and VII. Students who reported being bullied were predominantly from grade VII and VIII, whereas those who reported bullying others or being bully-victims were more frequently from grade IX. The proportion of participants in various bullying roles differ by the area of residence (χ^2^ (3) = 15.2, *p* = .02). A higher percentage of ‘no role’ group resided in rural areas, whereas victims, bullies, and bully-victims were more prevalent in urban areas. There was no significant association found based on gender or being a native Kannada speaker.

**Table 2:** Demographic characteristics of adolescents in bullying roles (*N* = 205)

Variables				Bullying roles						χ^2^
		Victim		Bully		Bully-Victim		No role		
		*n*	*%*	*n*	*%*	*n*	*%*	*n*	*%*	
Gender	Male	26	30.2	6	7.0	10	11.6	44	51.2	0.3
	Female	33	27.7	7	5.9	14	11.8	65	54.6	
School	Private	42	36.2	11	9.5	19	16.4	44	37.9	25.9**
	Government	17	19.1	2	2.2	5	5.6	65	73.3	
Grade	VII	32	36.8	3	3.4	6	6.9	46	52.9	27.8**
	VIII	16	25.4	3	4.8	3	4.8	41	65.1	
	IX	11	20.0	7	12.7	15	27.3	22	40.0	
Residence	Rural	19	21.6	2	2.3	7	8.0	60	68.2	15.2*
	Urban	40	34.2	11	9.4	17	14.5	49	41.9	
Kannada native language	Yes	27	23.3	5	4.3	14	12.1	70	60.3	7.2
	No	32	36.0	8	9.0	10	11.2	39	43.8	

*p < .05. **p < .001

### Types of Bullying

Across all the bullying roles, verbal bullying emerged as more prevalent than physical bullying. Among those who reported being bullied (*n* = 57), common forms of verbal bullying included being called names (54.4%), having jokes played on them (47.7%), and being made fun of (40.4%). Among individuals who admitted to bullying others (*n* = 13), common verbal bullying tactics reported were calling names (46.2%), making fun of others (46%), and playing jokes on them (46%). For those bully-victims who reported victimization (*n* = 24), prevalent forms of verbal bullying included being called names (62.5%), being made fun of (58.3%), and having jokes played on them (58.3%). On the other hand, bully-victims who also bullied others (*n* = 24) reported common behaviors such as calling names (37.5%) and making threats of doing harmful things to others (29.2%).

### Gender Differences in Bullying

In the victims’ group, no statistically significant difference was found between males and females in terms of physical bullying (*U* = 331.5, *p* = .25) and verbal bullying (*U* = 489.5, *p* = .14). Among bullies, no statistically significant difference was observed between males and females in terms of physical bullying (*U* = 15, *p* = .44) and verbal bullying (*U* = 14, *p* = .36). Physical and verbal bullying did not significantly differ in males and females among bully-victims who experienced victimization (Physical, *U* = 58, *p* = .50; Verbal, *U* = 57.5, *p* = .47) as well as those bullied others (Physical, *U* = 64.5, *p* = .75; Verbal, *U* = 60, *p* = .58). In the ‘no role’ group, no statistically significant differences were found between males and females regarding physical bullying (*U* = 51.5, *p* = .24) and verbal bullying (*U* = 46, *p* = .53).

### Perceptions of Students Regarding Bullying

Participant perceptions regarding the locations where bullying occurred, individuals involved, reasons for bullying/bullied, school knowledge and response regarding the bullying incidents are presented in Table 3.

**Table 3:** Bullying in various locations

Locations	Bullying roles									
	Victim		Bully		Bully-victim				No role	
					Bully others		Victimised			
	*n*	*%*	*n*	*%*	*n*	*%*	*n*	*%*	*n*	*%*
Classroom	29	50.9	4	30.8	11	45.8	12	20.0	3	16.7
Online/texting outside school	17	29.8	5	38.5	14	58.3	12	20.0	6	33.3
After school	13	22.8	5	38.5	9	37.5	5	8.3	3	16.7
Bathroom	7	12.3	1	7.7	3	12.5	2	3.3	3	16.7
Recess	7	12.3	3	23.1	3	12.5	7	11.7	3	16.7
Sports	7	12.3	1	7.7	4	16.7	4	16.7	1	5.6
Bus	6	10.5	1	7.7	4	16.7	5	8.3	0	0
Dances	4	7.0	1	7.7	4	16.7	2	3.3	4	22.2
Before school	2	3.5	1	7.7	2	8.3	3	5.0	0	0

Note. Percentages shown in the table indicates the number of respondents for each option

**Table 4:** Bullying via texting/online outside school

Texting/online outside school	Bullying roles									
	Victims (*n* = 17)		Bully (*n* = 5)			Bully-Victim			No role (*n* = 6)	
					Bully others (*n* = 15)		Victimised (*n* = 12)			
	*n*	*%*	*n*	*%*	*n*	*%*	*n*	*%*	*n*	*%*
Texting	**6**	**35.3**	1	20.0	3	20.0	4	33.3	0	0
Online gaming	5	29.4	1	20.0	4	26.7	4	33.3	1	16.7
WhatsApp	5	29.4	1	20.0	5	33.3	6	50.0	1	16.7
Facebook	3	17.6	**2**	**40.0**	5	33.3	4	33.3	0	0
Instagram	2	11.8	**2**	**40.0**	7	46.7	7	58.3	0	0
Email	1	5.9	1	20.0	1	6.7	1	8.3	1	16.7
Twitter	0	0	1	20.0	**1**	**6.7**	**0**	**0**	**3**	**50.0**

*Note. Percentages shown in the table indicates the number of respondents for each option*

#### Locations of Bullying

The primary locations reported by participants across bullying roles where bullying occurred included the classroom, online or texting after school, afterschool areas, and recess. Among those who reported victimization, a majority (35.3%) mentioned texting as the medium for bullying (see Table 4). On the other hand, participants classified as bullies identified Facebook (40%) and Instagram (40%) as platforms used for bullying others. For bully-victims, Instagram emerged as the major online medium for bullying. In comparison, the ‘no role’ group had fewer responses, with Twitter (50%) being reported as a platform where bullying occurred.

#### Persons Involved in Bullying

Participants identified individuals involved in bullying incidents based on the roles they assumed. Victims, bully-victims, and those in the ‘no role’ group reported individuals they perceived as potential bullies (Table 5), while bullies and bully-victims reported their potential victims (Table 6). Individuals who reported being bullied indicated that girls in the same grade (42.6%) and boys in the same grade (40.7%) were common perpetrators, followed by someone with many friends (27.8%) and someone physically strong (18.5%). Bully-victims who experienced victimization, as well as those in ‘no role’ group, identified girls and boys in the same grade (62.5% and 37.5%, respectively) as potential bullies, followed by someone with many friends (29.2%) and older boys (20.8%). In the ‘no role’ group, participants reported being bullied by individuals with many friends (25%), girls and boys in the same grade (18.8% each), and older boys (18.8%). Those who admitted to bullying others mentioned targeting both girls and boys in the same grade (33.3%) and individuals perceived as not smart (25%). Bully-victims who reported bullying others identified girls (52.2%) and boys (47.8%) in the same grade as their primary targets, followed by individuals perceived as popular and having many friends (21.7%).

**Table 5:** Bullies as reported by victims, bully-victims and no role groups

Bullies	Victims (*n* = 54)		Bully-Victims (*n* = 24)		No role (*n* = 16)	
	n	%	n	%	n	%
Older boys	9	16.7	5	20.8	3	18.8
Older girls	2	3.7	3	12.5	1	6.3
Younger boys	5	9.3	3	12.5	2	12.5
Younger girls	5	9.3	0	0	2	12.5
Boys in same grade	22	40.7	9	37.5	3	18.8
Girls in same grade	23	42.6	15	62.5	3	18.8
Someone who is strong	10	18.5	4	16.7	1	6.3
Someone who is weak	2	3.7	0	0	0	0
Someone who has friends	15	27.8	7	29.2	4	25.0
Someone who doesn’t have many friends	4	7.4	3	12.5	1	6.3
Someone who is popular	9	16.7	1	4.2	0	0
Someone who is not popular	4	7.4	2	8.3	1	6.3
Someone who is smart	9	16.7	4	5.5	0	0
Someone who is not smart	6	11.1	2	8.3	0	0
Someone who is an adult	9	16.7	1	4.2	0	0
My girlfriend/boyfriend	5	9.3	1	4.2	0	0
My sister	7	13.0	4	16.7	0	0
Someone who is in my group of friends	7	13.0	4	16.7	0	0

*Note. Percentages shown in the table indicates the number of respondents for each option*

**Table 6:** Victims as reported by bullies and bully-victims

Victims	Bullies (*n* = 12)		Bully-Victims (*n* = 23)	
	n	%	n	%
Older boys	**4**	**33.3**	3	13.0
Older girls	1	8.3	3	13.0
Younger boys	2	16.7	1	4.3
Younger girls	3	25.0	1	4.3
Boys in same grade	**4**	**33.3**	**11**	**47.8**
Girls in same grade	**4**	**33.3**	**12**	**52.2**
Someone who is strong	1	8.3	1	4.3
Someone who is weak	2	16.7	2	8.7
Someone who I don’t know	1	8.3	1	4.3
Someone I was interested but never went out with	1	8.3	1	4.3
Someone who is powerful	2	16.7	0	0
Someone who is not powerful	1	8.3	0	0
Someone who has friends	2	16.7	**5**	**21.7**
Someone who doesn’t have many friends	1	8.3	3	13.0
Someone who is popular	1	8.3	**5**	**21.7**
Someone who is not popular	2	16.7	1	4.3
Someone who is smart	1	8.3	3	13.0
Someone who is not smart	**3**	**25.0**	4	17.4
Someone who is an adult	0	0	1	4.3
My girlfriend/boyfriend	1	8.3	1	4.3
My brother	1	8.3	1	4.3
My sister	2	16.7	1	4.3

*Note. Percentages shown in the table indicates the number of respondents for each option*

#### Reasons for Bullying

Table 7 summarises the perceived reasons for bullying/victimisations across different bullying roles. Attributes such as funny-looking face and being fat was cited among all bullying roles. Victims identified reasons for being bullied, including having funny-looking face, displaying frequent anger, being fat, and achieving good grades. Bullies reported reasons for bullying such as funny-looking face, being fat, being in special education, and crying frequently. Bully-victims who also bullied others perceived reasons for bullying including funny-looking face, being fat, being tall or short, appearing weak, being skinny, getting angry, crying frequently, and specific ways of talking. Those who reported being victimized perceived reasons for victimization including being fat, being short, and having a funny-looking face. In the ‘no role’ group, the reasons for bullying included being in special education, being fat, appearing weak, having a funny-looking face, and coming from a financially disadvantaged family.

**Table 7:** Reasons for bullying/being victimised perceived across bullying roles

Reasons	Bullying roles									
	Victims (*n* = 49)		Bully (*n* = 13)		Bully-Victims (*n* = 22)				No role (*n* = 19)	
					Bully others		Victimised			
	*n*	*%*	*n*	*%*	*n*	*%*	*n*	*%*	*n*	*%*
Face looks funny	**10**	**20.4**	**5**	38.5	**6**	**27.3**	3	13.6	**3**	**15.3**
Fat	**9**	**18.4**	**5**	**38.5**	**5**	**22.7**	**6**	**27.3**	**4**	**21.1**
Skinny	1	2.0	2	15.4	3	13.6	1	4.5	1	5.3
Look too old	2	4.1	0	0	0	0	0	0	0	0
Look too young	2	4.1	2	15.4	1	4.5	0	0	0	0
Is a wimp	3	6.1	1	7.7	3	13.6	1	4.5	**4**	**21.1**
Friends are weird	6	12.2	1	7.7	0	0	2	9.1	1	5.3
Sick a lot	3	6.1	0	0	0	0	1	4.5	0	0
Disabled	2	4.1	2	15.4	0	0	0	0	0	0
Get good grades	**9**	18.4	1	7.7	1	4.5	3	13.6	1	5.3
Get bad grades	4	8.2	2	15.4	1	4.5	0	0	2	10.5
Where they live	1	2.0	0	0	1	4.5	1	4.5	1	5.3
Clothes they wear	1	2.0	1	7.7	0	0	0	0	1	5.3
Colour of skin	5	10.2	3	23.1	1	4.5	0	0	0	0
Country they are from	2	4.1	2	15.4	2	9.1	0	0	0	0
Different	1	2.0	1	7.7	0	0	0	0	0	0
Church they go to	0	0	0	0	0	0	0	0	0	0
Parents	1	2.0	0	0	1	4.5	0	0	1	5.3
Brother	2	4.1	1	7.7	1	4.5	1	4.5	0	0
Sister	5	10.2	1	7.7	1	4.5	0	0	0	0
Family is poor	3	6.1	0	0	0	0	1	4.5	**3**	**15.8**
Family has lot of money	2	4.1	2	15.4	1	4.5	0	0	0	0
Someone in the family has disability	2	4.1	1	7.7	0	0	0	0	0	0
Too tall	4	8.2	1	7.7	**4**	**18.2**	2	9.1	2	10.5
Too short	8	16.3	3	23.1	3	13.6	**4**	**18.2**	0	0
Special education	3	6.1	**5**	38.5	0	0	0	0	**5**	**26.3**
Angry a lot	**10**	20.4	3	23.1	3	13.6	2	9.1	1	5.3
Cry a lot	7	14.3	**4**	30.8	3	13.6	1	4.5	2	10.5
Cannot get along with	1	2.0	2	15.4	2	9.1	1	4.5	1	5.3
Gay	0	0	0	0	0	0	0	0	0	0
Way of talking	7	14.3	1	7.7	3	13.6	2	9.1	1	5.3
Act too much like boy	1	2.0	1	7.7	2	9.1	1	4.5	0	0
Act too much like girl	2	4.1	1	7.7	0	0	1	4.5	0	0
Other	5	10.2	1	7.7	**6**	**27.3**	**6**	**27.3**	2	10.5

*Note. Percentages shown in the table indicates the number of respondents for each option*

#### Knowledge and Response of the School Regarding Bullying

Victims (48%), bullies (54%) and bully-victims (those who bully others, 46%; those victimised, 54%) expressed uncertainty about whether the school staff and teachers were aware of the bullying incidents, thus were unsure about the staff’s response to the situation. Conversely, participants in the ‘no role’ group (56%) reported that the school staff and teachers were aware of bullying and responded effectively. A majority of the participants (69%) did not perceive bullying as a problem in their school; however, 74% of bully-victims identified bullying as a significant problem. Regardless of their bullying roles, almost half of the participants (48%) reported that their parents were unaware of the bullying incidents that they experienced.

### Attitude Towards Bullying

The analysis of pro-bullying attitudes among various bullying roles demonstrated marginal significance (*F* (3, 161) = 2.6, *p* = .05). A significant difference was observed between victims and bully-victims (*p* = .03), but not between other groups. There was a statistically significant difference in pro-bullying attitudes among the different grades (*F* (2,162) = 6.5, *p* = .002). Differences were identified between seventh and eighth graders (*p* = .06), and eighth and ninth graders (*p* = .04), but not between seventh and nineth graders (*p* = .81).

Table 8 shows pro-bullying attitude scores across gender, school type, residence, and native language. Male participants exhibited a higher pro-bullying attitude (28.7 ± 8.6) compared to female participants (25.2 ± 7.1) (*t* (163) = 2.8, *p* = .005). A significant difference was found between students from the private and government school (*t* (163) = 6.2, *p* < .001) with higher pro-bullying scores recorded among government school students (32.1 ± 5.9) compared to private school students (24.4 ± 7.7). Participants coming from rural residences had a higher pro-bullying attitude (32 ± 6.3) compared to their urban counterparts (24.1 ± 7.4) (*t* (163) = 6.6, *p* < .001). Participants with Kannada as their native language exhibited higher pro-bullying scores (28.2 ± 8) compared to those who did not (25.3 ± 7.7) (*t* (163) = 2.4, *p* = .01).

**Table 8:** Pro-bullying attitude

Variables		*n*	*M*	*SD*	*t*
Gender	Male	71	28.7	8.6	2.8*
	Female	94	25.2	7.1	
School	Private	115	24.4	7.7	6.2*
	Government	50	32.1	5.9	
Residence	Rural	54	32.0	6.3	6.6*
	Urban	111	24.1	7.4	
Kannada as native language	Yes	82	28.2	8.0	2.4*
	No	83	25.3	7.7	

*p < .05

## Discussion

### Bullying Roles

The study revealed that 47% of adolescents in seventh to ninth grades had experiences as victims, bullies, or bully-victims. This prevalence, while comparable to a systematic review by Thakkar et al. (2020), appears lower than the reported rates in other studies conducted in Karnataka (Chhabria et al., 2020; Ramya & Kulkarni, 2011; Ranjith et al., 2019). Notably, the rates of bullying perpetration (ranging from 7 to 31%) and victimization (ranging from 9 to 80%) in Indian studies vary widely, as reported by Thakkar et al. (2020). When contrasted with international studies, our findings of any victimization at 47% is higher compared to the prevalence reported in other regions, such as 23% (Chudal et al., 2022) and 22% (Chen & Elklit, 2017). Studies on global prevalence of bullying victimisation, such as the one conducted by Biswas et al. (2020) across 83 countries, demonstrated a pooled prevalence of 30.5% for bullying victimization among 12-17-year-olds, irrespective of income status. Prevalence varied significantly across regions, with the Eastern Mediterranean and African regions showing the highest rates (43-45%), while Europe demonstrated the lowest (8.4%). This wide variance in prevalence rates across and within countries may be attributed partly to the diverse methodologies and screening measures used in these studies. Varied questionnaire designs, encompassing single or multi-item assessments, differing definitions of bullying, and time frames (ranging from the past week to the last year) to measure bullying/victimization, contribute to the complexity of estimating prevalence. Moreover, cultural, and linguistic factors play a pivotal role in the reporting of bullying or victimization experiences (Zych et al., 2017). Culturally validated instruments that consider contextual relevance and linguistic understandability across diverse educational backgrounds, are crucial for obtaining accurate prevalence rates. Further research from different parts of the world, particularly from developing countries, is imperative to gain comprehensive understanding of school bullying (Biswas et al., 2020).

### Gender Differences in Bullying

Our study revealed an equal involvement of both boys and girls in bullying, aligning with the findings of Kshirsagar et al. (2007) in Maharashtra. However, contrary evidence from various other studies suggests a higher involvement of boys in bullying compared to girls (Armitage, 2021; Chen & Elklit, 2017; Malhi et al., 2014, 2015; Munni & Malhi, 2006; Patel et al., 2017; Patel et al., 2020; Ramya & Kulkarni, 2011; Sethi et al., 2019). Moreover, our study into pro-bullying attitudes among genders showed that boys tend to hold more favorable attitudes towards bullying compared to girls. This is similar to the findings of Rigby (1997), who suggested that attitudes may serve as a precursor to the frequency with which adolescents engage in bullying behaviour. This difference in attitudes may potentially stem from gender socialisation and entrenched gendered interaction patterns existing within families and schools, i.e., socially accepted norm portrays boys as more prone to aggression, where having a violent attitude is sometimes considered normative (Rosen & Nofziger, 2018). Our study found that both genders perceived girls and boys in the same grade in potential bullying roles, in addition to reports of older boys bullying younger students, targeting individuals considered ‘not smart,’ popular peers, and those with many friends. Our study did not find gender differences in verbal or physical bullying or victimization, in contrast to studies that showed higher rates of physical bullying and victimization among boys, whereas girls are more likely to report verbal or relational bullying and victimization (Pepler et al., 2008; Menesini & Salmivalli, 2017). The diminishing gender gap for these behaviour demands widespread scrutiny and further investigation in this area (Malik & Mehta, 2016).

### Types of Bullying

Our examination of bullying types among adolescents revealed that, like most of the other studies done in India (Chhabria et al., 2020; Patel et al., 2017; Rana et al., 2020; Thakkar et al., 2020), verbal bullying emerged as the frequently reported form in our sample. Instances of name calling, being made fun of, and playing jokes were frequent experiences observed across bullying roles. This pattern aligns with previous research findings (Kshirsagar et al., 2007; Patel et al., 2020; Ramya & Kulkarni, 2011). Despite its seemingly innocuous nature, verbal bullying hurts, and causes more enduring damage to the individual than other forms. Many studies have associated verbal bullying with adverse effects on mental health, including low self-esteem, conduct problems, depression, anxiety, and suicidal tendencies (Özdemir & Stattin, 2011; Malhi et al., 2014). Furthermore, it can result in educational consequences such as school absenteeism and anticipation of failure. Children frequently involved in bullying have an increased risk of adult adversities (Armitage, 2021).

### Bullying Profile

In our study, there were a higher prevalence of bullying with more victims, bullies, and bully-victims in the private school. This discrepancy in prevalence rates between school types might be attributed to the lesser homogeneity observed in socioeconomic status within private schools (Rana et al., 2020). Additionally, differences in bullying roles were observed across the school grades, with more victims in the seventh grade, and a higher prevalence of bullies and bully-victims in the ninth grade. This finding aligns with research indicating a decline in victimization during transition from grades, while bullying perpetration tends to remain stable within this population (Wang et al., 2016).

Most participants identified several locations where bullying commonly occurred, including classrooms, texting or online after school, in areas after regular school hours, and during recess (Malhi et al., 2015; Swearer & Cary, 2003). These locations shared a common characteristic of limited adult supervision. Studies consistently support this argument that bullying occurs in areas where staff presence appears low or when there is limited staff on duty. To prevent bullying, teachers should increase their supervision and take greater responsibility. Establishing the culture of respect, care, and safety while communicating that bullying is not acceptable in any form is pivotal (Gomba & Chen Tsai, 2012). Bullying via texting or online platform emerged as a prevalent form of harassment among the students, a trend echoed in a study conducted in high schoolers (Bhat et al., 2017). Social networking sites such as WhatsApp, Facebook, Instagram, and Twitter were commonly reported platforms where bullying occurred irrespective of bullying roles. Online gaming forums enabling multiplayer modes, were reported as the main platforms for cyberbullying. Implementing measures to prevent and monitor cyberbullying is imperative. Educating school administrators, parents, and students on parental controls, privacy and security settings, and the need for close monitoring of social media usage or developing clear policies and protocols specifically addressing cyberbullying could be instrumental (Espelage & Hong, 2017; Santre, 2022). Moreover, studies suggest that reduced computer usage among adolescents has been linked to decreased cyberbullying victimization (Hong et al., 2023).

### Reasons for Bullying

The primary reasons for being bullied or bullying others were often related to physical attributes such as weight, facial appearance, height, skin color, among others, followed by factors like academic performance, being in remedial or special education, and family economic status. Attribution theory suggests that individuals’ perceptions of events often shape their reactions more than the actual reality of those events (Pickens, 2005). Those perceived as different in any way were more likely to be bullied, with physical appearance being a major cause followed by skin color (Swearer & Cary, 2003; Patel et al., 2017; Nazir, 2019). Moreover, studies in India have shown an association between socioeconomic status (SES) and victimization. Children from lower SES backgrounds experienced more physical victimization, while those from higher SES backgrounds encountered more relational victimization (Malhi et al., 2015). Poor academic performance has also been associated with bully victimization in India (Patel et al., 2017). Additionally, Thakkar et al. (2020) found a relationship between religion and victimization, suggesting that non-Hindu children were more likely to be classified as victims as compared to Hindu children, although we did not record the religious affiliations of the children in our study. Prevention programs should emphasize the importance of inclusion and foster an environment that values diversity among students for healthy social development.

### Knowledge and Response of the School Regarding Bullying

School staff and teachers’ response to a bullying scenario from the perspective of a student is very important as they are more likely to pick up the very same attitude. Our study found a clear lack of awareness among students regarding the school’s response to bullying incidents, a trend consistent with findings from several studies conducted in India (Bhat et al., 2017; Kshirsagar et al., 2007; Malhi et al., 2015; Ramya & Kulkarni, 2011). This lack of awareness may stem from insufficient understanding of the school policies or terminologies related to bullying incidents. Many participants indicated that their parents were unaware of the bullying incidents that they faced, which is understandable when the students themselves do not recognize bullying as a prevalent problem in their environment. It underscores the necessity for clear communication among school staff, consistent reporting procedures, and instilling trust in their ability to address such incidents. Empowering staff through comprehensive training to effectively handle and prevent bullying is crucial, as they serve as pivotal sources of support for students.

The Government of India has taken measures to combat bullying and cyberbullying in schools by implementing mandatory anti-ragging committees and initiating cyber-safety awareness campaigns in certain states (Kaur & Saini, 2023). Additionally, legal provisions have been established to address online offenses (Kaur & Saini, 2023). However, there is currently no standardized nationwide anti-bullying program in schools. Instead, there are ongoing efforts to develop specific multicomponent intervention programs tailored for schools in India (e.g. Rana et al., 2022).

### Limitations

The present study had several limitations. Firstly, its cross-sectional nature limits the ability to establish causal relationships among the variables. Secondly, due to the small sample size, gender differences within the profiles across bullying roles could not be thoroughly examined. Additionally, reliance on self-reported data without external validation, particularly concerning attitudes and perceptions, may present biases, as children tend to underestimate their aggressive behavior while endorsing more prosocial behavior in self-reports (Salmivalli et al., 1996). Moreover, the study was conducted in only two co-educational schools in Udupi taluk, Karnataka, which limits generalizability. Furthermore, the absence of an assessment related to functional outcomes limits our understanding of the broader impact of bullying.

## Conclusions

The current findings indicated the adolescent involvement in various forms of bullying, requiring attention from the concerned authorities. Studying the perceptions on locations, individuals, reasons, and attitudes towards bullying, contributes to the sparse literature within the Indian context. The complexity inherent in the phenomenon of bullying phenomenon was apparent as it extends beyond behavioral aspects, including perceptions and attitudes (Swearer, 2003). Schools are optimal settings for developing, implementing, and investigating bullying prevention programs. Our findings suggests, bullying prevention programs should address the diverse roles individuals assume in bullying scenarios, their perceptions and attitudes towards bullying, adequate teacher and staff trainings to equip them with resources, and enabling parental supervision to promote a culture free from bullying. Schools must partner with researchers and mental health advocates to help plan, develop, and guide prevention and intervention programs (Swearer, 2003), adopt antibullying policies specific to their ecology, and communicate these policies to all school members for effective implementation.

## References

[CR1] Armitage, R. (2021). Bullying in children: Impact on child health. *BMJ Paediatrics Open*, *5*(1), e000939. 10.1136/BMJPO-2020-000939. 33782656 10.1136/bmjpo-2020-000939 PMC7957129

[CR2] Bhat, C. S., Ragan, M. A., Selvaraj, P. R., & Shultz, B. J. (2017). Online bullying among high-school students in India. *International Journal for the Advancement of Counselling*, *39*(2), 112–124. 10.1007/s10447-017-9286-y.

[CR3] Biswas, T., Scott, J. G., Munir, K., Thomas, H. J., Huda, M. M., Hasan, M. M., et al. (2020). Global variation in the prevalence of bullying victimisation amongst adolescents: Role of peer and parental supports. *EClinicalMedicine*, *20*, 100276. 10.1016/j.eclinm.2020.100276. 32300737 10.1016/j.eclinm.2020.100276 PMC7152826

[CR4] Chen, Y. Y., & Elklit, A. (2017). Exposure to bullying among adolescents across nine countries. *Journal of Child & Adolescent Trauma*, *11*(1), 121–127. 10.1007/s40653-017-0172-x. 32318143 10.1007/s40653-017-0172-x PMC7163880

[CR5] Chhabria, M. S., Rao, A., Rao, C., & Somashekar, A. R. (2020). Prevalence and forms of bullying perpetration and victimization in Indian adolescents. *International Journal of Medicine and Public Health*, *10*(4), 213–216. 10.5530/ijmedph.2020.4.45.

[CR6] Chudal, R., Tiiri, E., Brunstein Klomek, A., Ong, S. H., Fossum, S., Kaneko, H., & Eurasian Child Mental Health Study (EACMHS) Group. (2022). Victimization by traditional bullying and cyberbullying and the combination of these among adolescents in 13 European and Asian countries. *European Child & Adolescent Psychiatry*, *31*(9), 1391–1404. 10.1007/s00787-021-01779-6. 33884501 10.1007/s00787-021-01779-6 PMC9402766

[CR7] Denny, S., Peterson, E. R., Stuart, J., Utter, J., Bullen, P., Fleming, T., Ameratunga, S., Clark, T., & Milfont, T. (2015). Bystander intervention, bullying, and victimization: A multilevel analysis of New Zealand high schools. *Journal of School Violence*, *14*(3), 245–272. 10.1080/15388220.2014.910470.

[CR8] Eastman, M., Foshee, V., Ennett, S., Sotres-Alvarez, D., Reyes, H. L. M., Faris, R., & North, K. (2018). Profiles of internalizing and externalizing symptoms associated with bullying victimization. *Journal of Adolescence*, *65*, 101–110. 10.1016/j.adolescence.2018.03.007. 29573643 10.1016/j.adolescence.2018.03.007 PMC5932115

[CR9] Espelage, D. L., & Hong, J. S. (2017). Cyberbullying prevention and intervention efforts: Current knowledge and future directions. *Canadian Journal of Psychiatry*, *62*(6), 374–380. 10.1177/0706743716684793. 28562094 10.1177/0706743716684793 PMC5455869

[CR10] Evans-Lacko, S., Takizawa, R., Brimblecombe, N., King, D., Knapp, M., Maughan, B., & Arseneault, L. (2017). Childhood bullying victimization is associated with use of mental health services over five decades: A longitudinal nationally representative cohort study. *Psychological Medicine*, *47*(1), 127–135. 10.1017/S0033291716001719. 27677437 10.1017/S0033291716001719

[CR11] Ferrara, P., Franceschini, G., Villani, A., & Corsello, G. (2019). Physical, psychological and social impact of school violence on children. *Italian Journal of Pediatrics*, *45*(1), 76. 10.1186/s13052-019-0669-z. 31248434 10.1186/s13052-019-0669-z PMC6598284

[CR12] Gabrielli, S., Rizzi, S., Carbone, S., & Piras, E. M. (2021). School interventions for bullying-cyberbullying prevention in adolescents: Insights from the UPRIGHT and CREEP projects. *International Journal of Environmental Research and Public Health*, *18*(21), 11697. 10.3390/ijerph182111697. 34770212 10.3390/ijerph182111697 PMC8583537

[CR13] Gomba, C., & Chen Tsai, K. (2012). Effects of bullying in schools: The teachers’ perspectives. *Journal of Society and Communication*, 161–179.

[CR14] Gómez-López, M., Viejo, C., & Ortega-Ruiz, R. (2019). Psychological well-being during adolescence: Stability and association with romantic relationships. *Frontiers in Psychology*, *10*, 1772. 10.3389/fpsyg.2019.01772. 31428023 10.3389/fpsyg.2019.01772 PMC6688553

[CR15] Hinduja, S., & Patchin, J. W. (2010). Bullying, cyberbullying, and suicide. *Archives of Suicide Research*, *14*(3), 206–221. 10.1080/13811118.2010.494133. 20658375 10.1080/13811118.2010.494133

[CR16] Hong, J. S., Wang, M., Negi, R., Voisin, D. R., Takahashi, L. M., & Iadipaolo, A. (2023). Less computer access: Is it a risk or a protective factor for cyberbullying and face-to-face bullying victimization among adolescents in the United States? *Behavioral Sciences (Basel Switzerland)*, *13*(10), 834. 10.3390/bs13100834. 37887484 10.3390/bs13100834 PMC10603963

[CR17] Kaur, M., & Saini, M. (2023). Indian government initiatives on cyberbullying: A case study on cyberbullying in Indian higher education institutions. *Education and Information Technologies*, *28*(1), 581–615. 10.1007/s10639-022-11168-4. 35814802 10.1007/s10639-022-11168-4 PMC9251041

[CR18] Kelly, E. V., Newton, N. C., Stapinski, L. A., Slade, T., Barrett, E. L., Conrod, P. J., & Teesson, M. (2015). Suicidality, internalizing problems and externalizing problems among adolescent bullies, victims and bully-victims. *Preventive Medicine*, *73*, 100–105. 10.1016/j.ypmed.2015.01.020. 25657168 10.1016/j.ypmed.2015.01.020

[CR19] Kshirsagar, V. Y., Agarwal, R., & Bavdekar, S. B. (2007). Bullying in schools: Prevalence and short-term impact. *Indian Pediatrics*, *44*(1), 25–28. 17277427

[CR20] Malhi, P., Bharti, B., & Sidhu, M. (2014). Aggression in schools: Psychosocial outcomes of bullying among Indian adolescents. *Indian Journal of Pediatrics*, *81*(11), 1171–1176. 10.1007/s12098-014-1378-7. 24659440 10.1007/s12098-014-1378-7

[CR21] Malhi, P., Bharti, B., & Sidhu, M. (2015). Peer victimization among adolescents: Relational and physical aggression in Indian schools. *Psychological Studies*, *60*(1), 77–83. 10.1007/s12646-014-0283-5.

[CR22] Malik, A., & Mehta, M. (2016). Bullying among adolescents in an Indian school. *Psychological Studies*, *61*(3), 220–232. 10.1007/s12646-016-0368-4.

[CR23] Menesini, E., & Salmivalli, C. (2017). Bullying in schools: The state of knowledge and effective interventions. *Psychology Health & Medicine*, *22*(Sup1), 240–253. 10.1080/13548506.2017.1279740. 10.1080/13548506.2017.1279740 28114811

[CR24] Menken, M. S., Isaiah, A., Liang, H., Rivera, P. R., Cloak, C. C., Reeves, G., Lever, N. A., & Chang, L. (2022). Peer victimization (bullying) on mental health, behavioral problems, cognition, and academic performance in preadolescent children in the ABCD Study. *Frontiers in Psychology*, *13*, 925727. 10.3389/fpsyg.2022.925727. 36225678 10.3389/fpsyg.2022.925727 PMC9549775

[CR25] Nazir, T. (2019). Prevalence of school bullying in higher secondary school students and myths related to bullying among students. *Journal of Advances and Scholarly Researches in Allied Education*, *16*(4), 435–439.

[CR26] Özdemir, M., & Stattin, H. (2011). Bullies, victims, and bully-victims: A longitudinal examination of the effects of bullying‐victimization experiences on youth well‐being. *Journal of Aggression Conflict and Peace Research*, *3*(2), 97–102.

[CR27] Patel, H. A., Varma, J., Shah, S., Phatak, A., & Nimbalkar, S. M. (2017). Profile of bullies and victims among urban school-going adolescents in Gujarat. *Indian Pediatrics*, *54*(10), 841–843. 28699613 10.1007/s13312-017-1146-7

[CR28] Patel, V., Varma, J., Nimbalkar, S., Shah, S., & Phatak, A. (2020). Prevalence and profile of bullying involvement among students of rural schools of Anand, Gujarat, India. *Indian Journal of Psychological Medicine*, *42*(3), 268–273. 10.4103/IJPSYM.IJPSYM_172_19. 32612332 10.4103/IJPSYM.IJPSYM_172_19 PMC7320736

[CR29] Pepler, D., Jiang, D., Craig, W., & Connolly, J. (2008). Developmental trajectories of bullying and associated factors. *Child Development*, *79*(2), 325–338. 10.1111/j.1467-8624.2007.01128.x. 18366426 10.1111/j.1467-8624.2007.01128.x

[CR30] Pickens, J. (2005). Attitudes and perceptions. In N. Borkowski (Ed.), *Organizational behavior in health care* (pp. 43–75). Jones and Bartlett.

[CR31] Radliff, K., Hall, J., & Ökten, M. (2018). School bullying. In T. Shackelford, & V. Weekes-Shackelford (Eds.), *Encyclopedia of evolutionary psychological science*. Springer.

[CR32] Ramya, S. G., & Kulkarni, M. L. (2011). Bullying among school children: Prevalence and association with common symptoms in childhood. *Indian Journal of Pediatrics*, *78*(3), 307–310. 10.1007/s12098-010-0219-6. 20960076 10.1007/s12098-010-0219-6

[CR33] Rana, M., Gupta, M., Malhi, P., Grover, S., & Kaur, M. (2020). Prevalence and correlates of bullying perpetration and victimization among school-going adolescents in Chandigarh, North India. *Indian Journal of Psychiatry*, *62*(5), 531–539. 10.4103/psychiatry.IndianJPsychiatry_444_19. 33678834 10.4103/psychiatry.IndianJPsychiatry_444_19 PMC7909033

[CR34] Rana, M., Gupta, M., Malhi, P., Grover, S., & Kaur, M. (2022). Designing a multi-component ‘Stop bullying-school intervention program’ in Chandigarh, a north Indian Union Territory. *Global Health Promotion*, *29*(2), 68–77. 10.1177/17579759211021061. 34159858 10.1177/17579759211021061

[CR35] Ranjith, P. J., Jayakumar, C., Kishore, M. T., Binukumar, B., & Bhaskar, A. (2019). Association between bullying, peer victimization and mental health problems among adolescents in Bengaluru, India. *Indian Journal of Social Psychiatry*, *35*(3), 207–212. 10.4103/ijsp.ijsp_6_19.

[CR36] Rigby, K. (1997). Attitudes and beliefs about bullying among Australian school children. *Irish Journal of Psychology*, *18*(2), 202–220. 10.1080/03033910.1997.10558140.

[CR37] Rosen, N. L., & Nofziger, S. (2018). Boys, bullying, and gender roles: How hegemonic masculinity shapes bullying behaviour. *Gender Issues*, *36*(3), 295–318. 10.1007/s12147-018-9226-0.

[CR38] Salmivalli, C., & Voeten, M. (2004). Connections between attitudes, group norms, and behaviour in bullying situations. *International Journal of Behavioral Development*, *28*(3), 246–258. 10.1080/01650250344000488.

[CR39] Salmivalli, C., Lagerspetz, K., Björkqvist, K., Österman, K., & Kaukiainen, A. (1996). Bullying as a group process: Participant roles and their relations to social status within the group. *Aggressive Behavior*, *22*(1), 1–15. 10.1002/(SICI)1098-2337(1996)22:1%3C;1::AID-AB1%3E;3.0.CO;2-T.

[CR40] Santre, S. (2022). Cyberbullying in adolescents: A literature review. *International Journal of Adolescent Medicine and Health*, *35*(1), 1–7. 10.1515/ijamh-2021-0133. 35245420 10.1515/ijamh-2021-0133

[CR41] Sethi, S., Setiya, R., & Kumar, A. (2019). Prevalence of school bullying among school children in urban Rohtak, State Haryana, India. *Journal of Indian Association for Child and Adolescent Mental Health*, *15*(4), 13–28. 10.1177/0973134220190402.

[CR42] Swearer, S. M., & Cary, P. T. (2003). Perceptions and attitudes toward bullying in middle school youth: A developmental examination across the bully/victim continuum. *Journal of Applied School Psychology*, *19*(2), 63–79. 10.1300/J008v19n02_05.

[CR43] Swearer, S. M., Song, S. Y., Cary, P. T., Eagle, J. W., & Mickelson,W. T (2001). Psychosocial correlates in bullying and victimization. *Journal of Emotional Abuse*, *2*(2–3), 95–121. 10.1300/J135v02n02_07.

[CR44] Takizawa, R., Maughan, B., & Arseneault, L. (2014). Adult health outcomes of childhood bullying victimization: Evidence from a five-decade longitudinal British birth cohort. *The American Journal of Psychiatry*, *171*(7), 777–784. 10.1176/appi.ajp.2014.13101401. 24743774 10.1176/appi.ajp.2014.13101401

[CR46] Thakkar, N., van Geel, M., Malda, M., Rippe, R. C. A., & Vedder, P. (2020). Bullying and psychopathic traits: A longitudinal study with adolescents in India. *Psychology of Violence*, *10*(2), 223–231. 10.1037/vio0000277.

[CR45] Thakkar, N., van Geel, M., & Vedder, P. (2021). A systematic review of bullying and victimization among adolescents in India. *International Journal of Bullying Prevention*, *3*, 253–269. 10.1007/s42380-020-00081-4.

[CR47] Touloupis, T., & Athanasiades, C. (2022). Evaluation of a cyberbullying prevention program in elementary schools: The role of self-esteem enhancement. *Frontiers in Psychology*, *13*, 980091. 10.3389/fpsyg.2022.980091. 36211905 10.3389/fpsyg.2022.980091 PMC9537075

[CR48] van Goethem, A. A., Scholte, R. H., & Wiers, R. W. (2010). Explicit- and implicit bullying attitudes in relation to bullying behavior. *Journal of Abnormal Child Psychology*, *38*(6), 829–842. 10.1007/s10802-010-9405-2. 20352324 10.1007/s10802-010-9405-2

[CR49] Wang, W., Brittain, H., McDougall, P., & Vaillancourt, T. (2016). Bullying and school transition: Context or development? *Child Abuse & Neglect*, *51*, 237–248. 10.1016/j.chiabu.2015.10.004. 26522183 10.1016/j.chiabu.2015.10.004

[CR50] Wolke, D., & Lereya, S. T. (2015). Long-term effects of bullying. *Archives of Disease in Childhood*, *100*(9), 879–885. 10.1136/archdischild-2014-306667. 25670406 10.1136/archdischild-2014-306667 PMC4552909

[CR51] Zych, I., Farrington, D. P., Llorent, V. J., & Ttofi, M. M. (2017). School bullying in different countries: Prevalence, risk factors, and short-term outcomes. In I. Zych, D. P. Farrington, V. J. Llorent, & M. M. Ttofi (Eds.), *Protecting children against bullying and its consequences*. Springer.

